# Visual Evoked Potentials for the Detection of Diabetic Retinal Neuropathy

**DOI:** 10.3390/ijms24087361

**Published:** 2023-04-17

**Authors:** Gen Miura

**Affiliations:** Department of Ophthalmology and Visual Science, Chiba University Graduate School of Medicine, Inohana 1-8-1, Chuo-ku, Chiba 260-8677, Japan; gmiura2@chiba-u.jp

**Keywords:** visual evoked potential, diabetes mellitus, diabetic retinopathy

## Abstract

Visual evoked potentials (VEP) are visually evoked signals that extract electroencephalographic activity in the visual cortex that can detect retinal ganglion cells, optic nerves, chiasmal and retrochiasmal dysfunction, including optic radiations, and the occipital cortex. Because diabetes causes diabetic retinopathy due to microangiopathy and neuropathy due to metabolic abnormalities and intraneural blood flow disorders, assessment of diabetic visual pathway impairment using VEP has been attempted. In this review, evidence on the attempts to assess the visual pathway dysfunction due to abnormal blood glucose levels using VEP is presented. Previous studies have provided significant evidence that VEP can functionally detect antecedent neuropathy before fundus examination. The detailed correlations between VEP waveforms and disease duration, HbA1c, glycemic control, and short-term increases and decreases in blood glucose levels are evaluated. VEP may be useful for predicting postoperative prognosis and evaluating visual function before surgery for diabetic retinopathy. Further controlled studies with larger cohorts are needed to establish a more detailed relationship between diabetes mellitus and VEP.

## 1. Introduction

According to the International Diabetes Federation report for 2021, approximately 537 million individuals are living with diabetes, and it is projected to reach 783 million by 2045. Diabetic ocular complications are asymptomatic at early stages and cause visual impairment unless diagnosed early and treated [1].

Electrophysiological testing is an objective noninvasive method for evaluating target function. The electroretinogram (ERG), which is an important electrophysiological test in the field of ophthalmology, is capable of evaluating the functions of various retinal cells and has contributed to deepening our understanding of retinal neuronal damage caused by diabetes.

Comprehensive reviews of ERG waveform alterations in patients with diabetes have previously been conducted [2,3,4,5]. As examples, normal a- and b-waves or slightly reduced amplitude in dark-adapted ERGs in patients with diabetes [6,7], b-wave amplitude reduction and latency increase in both scotopic and photopic ERGs [8], reduced oscillatory potentials amplitude in scotopic and multifocal ERG [9,10,11,12], photopic negative response amplitude reduction [13,14], decrease in amplitude and prolongation of latency in flicker ERG [15,16,17,18], decreased scotopic b-wave amplitude, decreased flicker ERG amplitude, and increased a-wave latency in light-adapted ERG in patients with proliferative diabetic retinopathy (DR) who underwent panretinal photocoagulation [19], and the usefulness of multifocal ERG for predicting the progression of vascular abnormalities due to diabetes [20,21] have been reported.

As mentioned above, there have been many reports on diabetes and ERG. However, there are few reviews on the relationship between diabetes and visual evoked potential (VEP). VEP is the total visually evoked potential extracted from electroencephalography activity in the visual cortex recorded from the scalp electrodes, which is a noninvasive objective test aimed at assessing nerve impulse transmission along the visual pathway from the retinal photoreceptor to the calcarine cortex via the optic nerve.

Since the visual cortex is primarily activated by the central visual field, VEP reflects the function of the central visual field at all levels of the visual pathway, including the retinal ganglion cells, optic nerve, optic radiation, and occipital lobe. Therefore, although this depends on the settings described later, VEP generally reflects the function of the macula to a relatively large extent.

Light responses are recorded in the visual cortex by various light stimuli projected onto the retina, which maintains a constant average brightness while reversing the contrast over time. Three typical patterns have been widely used in relevant studies: sine-wave gratings, square-wave gratings, and checkerboards. The response has been evaluated based on the amplitude, latency, morphology, and transoccipital distribution of the obtained waveforms.

A systematic review is required to understand how VEP has been applied in diabetes research and how its results have been interpreted. In this review, we summarize the principles and rationale for VEP and present findings from recent clinical studies to assess the neurological dysfunction in patients with diabetes. We then review the current status and usefulness of these studies as well as the strengths and limitations of the VEP examination.

## 2. Stimulus Conditions and Waveforms

### 2.1. Full-Field VEP

**Figure 1:**
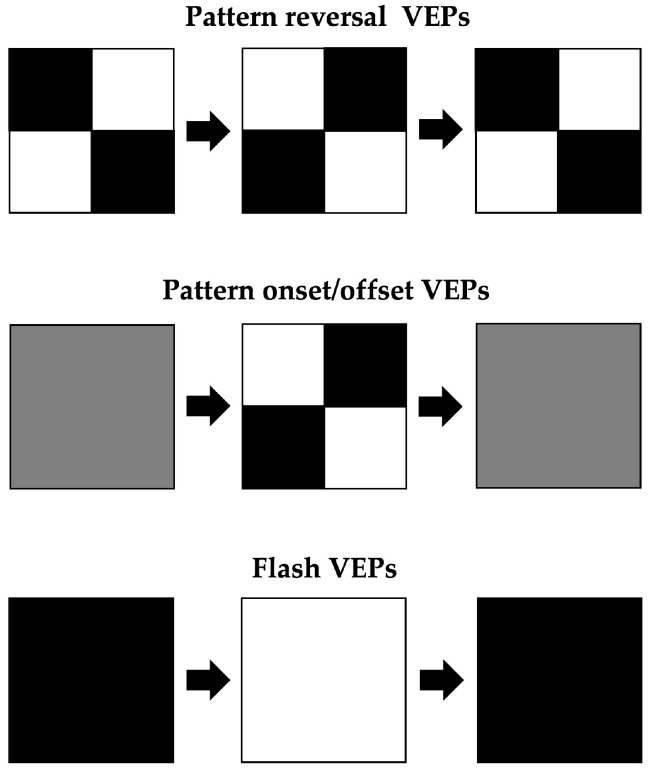
Schematic illustration of pattern reversal, pattern onset/offset and flash VEPs stimuli.

Several stimulus patterns have been used for VEP assessment. To enable evaluation in a uniform setting even in different facilities and regions, the International Society for Clinical Electrophysiology of Vision (ISCEV) has introduced standards for eliciting and recording VEP [22]. The ISCEV proposes three stimulation methods: pattern reversal, pattern onset/offset, and flash VEPs (Figure 1).

Pattern reversal VEPs are elicited by checkerboard stimuli with large, 1° (acceptable range of 0.8° to 1.2°), and small, 0.25° (0.2° to 0.3°), checks. Pattern reversal VEP has less variation in waveforms and latencies due to individual differences between subjects than VEP using other stimulation conditions and has excellent reproducibility. A typical pattern reversal VEP waveform consists of a negative wave N75, a positive wave P100, and a negative wave N135. The P100 waveform is generated in the striate and peristriate occipital cortex due to the activation of the primary visual cortex and the discharge of thalamocortical fibers. N70 reflects the activity of the fovea and primary visual cortex, whereas N145 reflects the activity of the visual association cortex.

Pattern onset/offset VEPs are elicited by checkerboard stimuli with large (1°; 0.8° to 1.2°) and small (0.25°; 0.2° to 0.3°) checks. This type of VEP reflects the function of the macular pathway and is more effective in detecting malingering and for use in patients with nystagmus. A typical pattern onset/offset VEP is composed of a positive wave C1, a negative wave C2, and a positive wave C3. C1 is believed to originate from multiple visual areas and has a predominant contribution from the V1 primary striate visual cortex in the early part of the waveform. C2 arises from the dorsal and ventral extra-striate, and C3 arises from the posterior parietal cortex, etc.

Flash VEPs are elicited by a flash (brief luminance increment) that subtends a visual field of at least 20° recorded by a strobe or LED flash stimulator. This stimulation is useful in animal studies where poor optical quality, uncooperativeness, young children, or poor visual acuity make pattern stimulation inappropriate. A typical waveform of flash VEP consists of a negative wave N1, positive wave P1, negative wave N2, positive wave P2, negative wave N3, and positive wave P3. The positive peak (P2) and leading negative peak (N2) are the easiest waveforms to measure and are, therefore, commonly used in clinical evaluation. Previous studies suggest that the transient luminance flash VEP waveforms primarily arise from the activity of the striate and extrastriate cortex.

Pattern onset/offset stimulation and flash VEP have large individual waveform differences but are useful for detecting intracranial pathway dysfunction and for patients with interocular differences [23,24,25,26].

Although the examination is usually performed without using color, attempts have been made to evaluate parallel parvocellular and koniocellular visual pathways using red–green or blue–yellow stimuli, and its usefulness has been reported in demyelinating diseases [27,28,29], Leber’s hereditary optic neuropathy [30], glaucoma [31], Parkinson’s disease [32], and congenital color blindness [33].

### 2.2. Multifocal VEP

Conventional (full-field) VEP waveforms provide the summed response of all stimulated neuronal elements and are greatly affected by the macular region owing to cortical overrepresentation [34]. Specifically, 65% of the VEP responses are estimated to represent the central 2° of the visual field [35]. The small check size commonly used for pattern stimulation is another factor that tends to bias the central responses [36]. The VEP response does not provide local information; thus, it has the disadvantage of not being able to identify localized damage [22].

In 1994, Baseler reported a method for measuring VEPs using multifocal stimulation to obtain local responses [37]. It consisted of 60 black-and-white checkered sectors and VEPs using pseudo-randomized m-binary sequences termed multifocal visual evoked potentials (mfVEP). Similar to VEP, mfVEP is an objective test; thus, it is not affected by physical or psychological factors, age, or sex [38]. MfVEP was initially applied, modified, and refined to investigate cortical responses in glaucoma and demyelinating diseases [39,40,41]. For example, the amplitude of mfVEP correlated with the Humphrey field analyzer 24-2 visual field loss [42], glaucoma progression correlated with the mfVEP latency [43,44,45], mfVEP was more effective than Standard Automated Perimetry in monitoring mild damage to ganglion cells [46], mfVEP is more reliable for reproducibility than the Humphrey field analyzer [47], and mfVEP is useful in diagnosing diseases such as ischemic and compressive optic neuropathy, optic neuritis, and multiple sclerosis [48,49].

## 3. Waveform Interpretation

VEP represents the electrical signal generated at the level of the striatal cortex by the combined activity of postsynaptic potentials in response to visual stimuli [50]. Consequently, its magnitude (“amplitude”) and timing (“latency”) are subject to pathological changes along the entire visual pathway.

Multiple sclerosis studies have shown that the amplitude of the VEP reflects the number of functional fibers along the visual pathway and is determined by the severity of neuropathy in the acute phase of the disease and by axonal degeneration later in the disease [51].

Latency is related to the conduction velocity. The extent of axonopathy is thought to be correlated with the delay in the VEP arrival to the visual cortex, that is, latency delay, since conduction delay affects the demyelinated portion of the axon.

Therefore, in patients with optic neuritis, a significant correlation was reported between the length of the optic nerve lesions and the relative latency delay of the VEP from the corresponding ocular stimuli [52]. A similar relationship has been reported in animal studies [53]. In addition, a significant correlation between VEP latency delay and optic radiation fiber lesion volume in patients with multiple sclerosis has been reported [54].

Currents from all excitatory nerve membranes contribute to the overall VEP response [55], and waveforms are contaminated by other voltage-gated neural events. Therefore, in interpreting global electrical responses such as VEPs, consideration must be given to the spatial and temporal arrangement and the structure of the responding neurons. This factor may lead to discrepancies and difficulties in interpretation when reviewing the relationship between diabetes mellitus (DM) and VEP waveforms.

## 4. Evaluation of Diabetic Retinopathy

DR is the most common microvascular complication of diabetes and the major cause of visual acuity loss and blindness in working-age adults [56]. DR is almost asymptomatic in the first 10 years after the onset of diabetes [57]. This long-term absence of subjective symptoms contributes to the exacerbation of DR and delayed treatment. However, fundus photography, which is widely performed as a screening tool for DR, is often unable to detect early changes. Therefore, various modalities aimed at detecting early morphological and functional abnormalities in DR have been investigated. Specifically, changes due to early DR can be detected using optical coherence tomography (OCT), optical coherence tomography angiography (OCTA), contrast sensitivity, ERG, corneal confocal microscopy, and fundus perimeter.

Early changes in the retinal microvasculature and decreased deep capillary plexus perfusion density can be detected by OCTA in patients diagnosed with insulin-dependent diabetes mellitus (IDDM) but without DR [58,59]. Patients with diabetes without DR have reduced retinal sensitivity at the fundus perimeter [60]. Eyes with diabetic neuropathy without DR had significantly thinner retinal nerve fiber layers and thicker retinal pigmented epithelium. There was a significant correlation between the retinal sensitivity as measured by the fundus perimeter MP-1 and diabetic neuropathy [61]. A significant reduction in corneal nerve fiber density, corneal nerve fiber length, and corneal nerve branch density using corneal confocal microscopy was reported in subjects with diabetes and no DR compared to healthy subjects [62,63,64]. In recent years, corneal nerve fiber density, corneal nerve fiber length, and corneal nerve branch density using corneal confocal microscopy have been suggested to correlate with HbA1c and the homeostatic model assessment of insulin resistance [65]. These studies show that changes in the ocular nerve structure due to DM occur even in the absence of ophthalmoscopic DR, which is clarified by an ERG, OCT, OCTA, corneal confocal microscopy, and fundus perimeter. Similarly, many studies have evaluated DM using VEP, which can objectively assess the function of the visual pathway.

## 5. Effects of DM and Abnormal Blood Glucose Levels on VEP Waveforms

### 5.1. Changes in Latency

Many studies on VEP in patients with DM have investigated the changes in latency. Specifically, the results showed that patients with DM had a significantly longer latency than the controls [66,67,68,69,70,71,72,73,74,75,76,77,78,79,80,81,82,83,84,85,86,87,88,89,90,91,92]. In addition, many studies have reported that a significant delay in latency was similarly observed in patients with DM who had not yet developed DR [34,69,70,72,76,82,93,94,95,96,97].

The previously reported delayed VEP latencies in patients with DM are thought to reflect delayed conduction along the optic nerve pathway. These results are consistent with studies using pattern ERGs [98] and retinocortical times [69].

These results suggest that VEP latency may be more sensitive to changes in the inner retinal and optic nerve pathways in the early stages of DM than fundus examination for the detection of ocular damage due to DM. The results of the survey on the relationship between VEP latency and blood glucose level, control status, duration of disease, and severity of retinopathy were reviewed.

### 5.2. Relationship between HbA1c, Disease Duration, and VEP Waveforms

Some reports have shown a significant correlation between VEP latency and the blood glucose level (hemoglobin A1c [HbA1c]) [81,92], while others have shown no correlation [67,82,83], and no clear conclusion has been reached.

What is the relationship between VEP latency and disease duration? Several studies have reported that VEP latency significantly correlates with disease duration [71,72,74,76,77,88,96,99]. On the other hand, there is also a report that there is no correlation between latency prolongation and disease duration [67,82,83,85]. Others have reported that latency is not correlated with age [67], HbA1c [82], or both [83], or with or without degenerative complications (neuropathy, nephropathy, or retinopathy) [85]. In addition, prolonged latencies were reported in patients with gestational DM with relatively short disease duration [100], and there were cases of IDDM with prolonged latencies and normal amplitudes in patients with disease duration <6 months [70,73].

These reports suggest that the close relationship between latency and disease duration is weak. Whether VEP latency can be an indicator of HbA1c, long-term control of blood glucose levels, or disease duration remains undetermined.

A negative correlation has been reported between latency and motor and sensory conduction velocities in all the examined nerves [67]. Gregori et al. showed that patients with DM and peripheral nerve damage had greater VEP abnormalities than patients without evidence of peripheral nerve involvement and that such abnormalities were associated with disease duration [101]. In another study, the mean P100 latency in young patients with IDDM was significantly increased bilaterally, and a significant correlation was observed between the P100 latency and the median value of the somatosensory evoked potential parameter [102].

The results of these studies suggest that central nervous system damage in young patients correlates with peripheral conduction disturbances. VEP may be more useful if peripheral nerve damage is considered when evaluating the relationship between the VEP waveform and indexes, such as HbA1c and disease duration.

### 5.3. Relationship between Short-Term Blood Glucose Level Fluctuations and VEP Waveforms

What kind of reports have there been about the relationship between VEP latency and relatively short-term fluctuations in blood glucose levels?

Several reports have shown that short-term improvements in blood glucose levels improved VEP waveform abnormalities [75,84,97,103]. In a study of newly diagnosed IDDM, improvements in blood glucose levels were associated with improved waveforms 6 months after diagnosis [84]. As a result of investigating the effect of short-term glycemic control, an improvement in the VEP waveform by 3-day glycemic control was reported [97]. However, their results showed no correlation between the blood glucose level reduction and waveform improvement.

Although it was a study using rats, it was also reported that streptozotocin-induced diabetic rats had significantly shorter latencies than rats that were not exercised [104].

These findings suggest that improving blood glucose levels may improve the VEP waveforms early after onset. On the other hand, there are also reports that glycemic control did not improve VEP abnormalities [73,76,85,86]. Algan et al. reported that prolonged latency did not correlate with the type of DM, disease duration, blood glucose level control, or degenerative complications (neuropathy, nephropathy, or retinopathy) [85].

There are also reports of the effect of short-term hyperglycemia or hypoglycemia on VEP waveforms. It has been reported that a rapid rise in blood glucose levels prolongs latency [105], and there is a significant correlation between short-term hyperglycemia and latency [100].

In contrast, a rapid rise in blood glucose levels did not prolong the latency in a study targeting patients with IDDM [106]. Additionally, hypoglycemia has been reported to result in prolonged latencies [107,108] or reduced amplitudes [78,108].

Conversely, some studies have reported that hypoglycemia does not affect VEP waveforms [109,110]. An investigation supporting the results showed that there was no change in the waveform before and after the tumor removal when the VEP was recorded in seven patients with chronic hypoglycemia due to insulinoma before and after the tumor removal [111].

Various results have been reported regarding the relationship between short-term changes in blood glucose levels and VEP waveforms, but strong evidence is still lacking.

### 5.4. Relationship between Surgical Intervention and VEP Waveforms

VEP waveforms are believed to be affected by surgical intervention.

After panretinal photocoagulation in patients with proliferative diabetic vitreoretinopathy and poor blood glucose control (HbA1c ≥ 10%), the VEP amplitude decreased in 48% of eyes, and latency was significantly prolonged in 75% of eyes [112]. However, in addition to the central damage due to DM, the causes of the VEP waveform changes reported in this study may include the effects of the redistribution of neurochemicals in the visual cortex following panretinal photocoagulation.

Attempts have also been made to investigate the effects of VEP on proliferative DR (PDR) after pars plana vitrectomy and to predict postoperative visual function. After pars plana vitrectomy in patients with advanced PDR and traction on the nasal side of the optic disc, pallor of the optic nerve head, and reduced visual acuity, 47% showed higher VEP amplitudes, and 35% showed a reduction in VEP latency [113]. In another report, dividing patients with PDR into two groups based on whether flash VEP latencies were shorter or longer than 100 ms, the patients with shorter latencies showed significantly greater improvement in postoperative visual function than patients with longer latencies [114].

Weinstein focused on the value of various temporal stimulus properties and consequently introduced flicker stimuli into VEP [115]. It has been used in several studies to predict visual function after vitrectomy.

Flash and flicker (5, 10, 20, 30 Hz) VEP were recorded in 245 eyes and showed that the flash VEP had no value. The presence of a response to a flicker stimulus of 10 Hz or higher indicated better functional recovery (*p* < 0.02). More eyes with an attached central retina showed a 30-Hz flicker response than eyes with central retinal detachment (*p* < 0.01). The study of flash and flicker VEP performed in complicated DR, including patients with proliferative vitreoretinopathy, showed that flicker VEP was a significant indicator of better postoperative visual function and eyes with an attached central retina. In contrast, the flash VEP study did not show any significant results that could serve as an index [116]. Vadrevu et al. investigated 44 diabetic eyes with vitreous hemorrhage and showed that the degree of 10 Hz flicker VEP abnormality was used to predict visual function after vitreous surgery or spontaneous resolution of vitreous hemorrhage, with an accuracy rate of 86%, reaching a statistically significant level [117].

Therefore, flicker VEP may have a predictive value before vitrectomy.

### 5.5. Effect of Blood Glucose Levels in Newborns, Infants, and Children

Investigating the effects of DM in neonates and children using VEP is also important [74,78,83,84,92,118,119,120,121].

Three-year-old offspring born to mothers with type 1 diabetes showed significantly prolonged VEP latencies [118].

The offspring of non-IDDM (NIDDM) parents showed a significant delay in VEP P100 latency compared with the controls. When compared within groups with NIDDM parents, offspring with both parents with diabetes showed significantly longer P100 latencies than those with one parent with diabetes [120].

Flash VEP in neonates of mothers with gestational diabetes between 37 and 41 weeks of gestation at birth was investigated, and their VEP latency was significantly prolonged compared with the controls. Additionally, P2 latency decreased significantly with increasing gestational and postnatal ages. This suggests that P2 latency may be a potential measure of cortical maturation [119].

In 2-month-old infants born to mothers with gestational diabetes, VEP latencies were significantly longer than those of the controls and were negatively associated with the Apgar scores [122].

These results suggest that VEP may detect DM-induced changes in neonates and infants.

### 5.6. Changes in Amplitude

Studies on DM and VEP waveforms have reported not only latency but also amplitude. Many reports have shown no change in VEP amplitude in patients with DM compared with the controls [80,84]. On the other hand, there are reports of significantly reduced VEP amplitudes in patients with DM or hyperglycemia [71,72,74,75,79,82,87,92,94,95,123].

A possible interpretation is that the impairment of functional fibers in the visual pathway caused by DM in some patients leads to reduced VEP amplitudes. However, there is also a report of a negative correlation between amplitude and age at diagnosis [92], and careful discussion of the relationship between VEP amplitude and DM is still necessary.

### 5.7. Multifocal VEP

Although there are not many reports examining mfVEP in patients with DM, results that are generally consistent with reports using full-field VEP have been reported.

Patients with diabetes and neuropathy reported a greater decrease in mfVEP amplitude than those without neuropathy, suggesting that mfVEP may assess optic neuropathy in patients with DR [89].

Significant mfVEP latency differences were found between the controls and all patients with diabetes, between the controls and the patients with diabetes without retinopathy, and between the controls and the patients with DR. In other words, mfVEP could detect DM-induced neurodegenerative changes from the retina to the central nervous system, even in patients without retinopathy. In the retinopathy group, latencies from zones with retinopathy were significantly longer than those without retinopathy (*p* = 0.016). However, there is little spatial association between the mfVEP abnormalities and non-PDR. The mfERG latency was more sensitive to the effects of diabetes than the mfVEP latency. The authors stated that this difference was due to the small sample size and problems with VEP measurements (body fat, muscle tension, and alpha waves) [90].

In a report comparing mfVEP in 18 patients having polyneuropathy with mfVEP in 14 diabetic and 10 nondiabetic patients without polyneuropathy, the amplitude of the mfVEP was significantly lower in both groups of DR patients than that in the healthy group of patients. mfVEP amplitudes, which reflect selected regions of visual function, were significantly reduced in the inferior nasal cavity of patients with neuropathy compared to those without neuropathy [91].

Regarding the usefulness of DM evaluation by mfVEP, it is still in the process of being clarified compared to research on glaucoma, and evidence based on a larger cohort and a sham-controlled trial is needed.

## 6. Conclusions

Previous VEP studies on DM have demonstrated the presence of antecedent neuropathy in patients with diabetes, including those without clinically evident ophthalmoscopic abnormalities or morphological changes. These studies provide significant evidence that VEP can functionally detect antecedent neuropathy before fundus examination.

The detailed correlations between VEP waveforms and disease duration, HbA1c, glycemic control, and short-term increases and decreases in blood glucose levels are controversial.

Flicker VEP may be useful for postoperative prognosis prediction and visual function evaluation before surgery for DR. Attempts have also been made to compensate for the difficulty of local retinal evaluation, which is a drawback of VEP, using mfVEP. Recent research has focused on the development of a new portable VEP device for easier testing [124,125].

Small sample sizes, heterogeneous study cohorts, and variability in the timing of glucose measurements are noted as limitations of previous studies on the correlation between DM and VEP. Further controlled studies with larger cohorts are needed to establish a more detailed relationship between DM and VEP.

Since VEP is almost the only tool that can detect dysfunction of the visual pathway from the retina and optic nerve to the visual cortex, VEP will continue to be important in the future.
